# Circumscribing Laser Cuts Attenuate Seizure Propagation in a Mouse Model of Focal Epilepsy

**DOI:** 10.1002/advs.202300747

**Published:** 2024-05-29

**Authors:** Seth Lieberman, Daniel A. Rivera, Ryan Morton, Amrit Hingorani, Teresa L. Southard, Lynn Johnson, Jennifer Reukauf, Ryan E. Radwanski, Mingrui Zhao, Nozomi Nishimura, Oliver Bracko, Theodore H. Schwartz, Chris B. Schaffer

**Affiliations:** ^1^ Meinig School of Biomedical Engineering Cornell University Ithaca NY 14853 USA; ^2^ College of Veterinary Medicine Cornell University Ithaca NY 14853 USA; ^3^ Statistical Consulting Unit Cornell University Ithaca NY 14853 USA; ^4^ Department of Neurological Surgery Weill Cornell Medicine of Cornell University New York NY 10065 USA; ^5^ Brain and Mind Research Institute Weill Cornell Medicine of Cornell University New York NY 10021 USA; ^6^ Department of Biology The University of Miami Coral Gables FL 33134 USA

**Keywords:** epilepsy, femtosecond infrared laser, focal seizures, surgery, treatment

## Abstract

In partial onset epilepsy, seizures arise focally in the brain and often propagate. Patients frequently become refractory to medical management, leaving neurosurgery, which can cause neurologic deficits, as a primary treatment. In the cortex, focal seizures spread through horizontal connections in layers II/III, suggesting that severing these connections can block seizures while preserving function. Focal neocortical epilepsy is induced in mice, sub‐surface cuts are created surrounding the seizure focus using tightly‐focused femtosecond laser pulses, and electrophysiological recordings are acquired at multiple locations for 3–12 months. Cuts reduced seizure frequency in most animals by 87%, and only 5% of remaining seizures propagated to the distant electrodes, compared to 80% in control animals. These cuts produced a modest decrease in cortical blood flow that recovered and left a ≈20‐µm wide scar with minimal collateral damage. When placed over the motor cortex, cuts do not cause notable deficits in a skilled reaching task, suggesting they hold promise as a novel neurosurgical approach for intractable focal cortical epilepsy.

## Graphical abstract

Blocking the spread of epileptic seizures in the brain can provide symptomatic relief for patients. Tightly‐focused infrared femtosecond laser pulses can produce micrometer‐sized cuts deep inside tissue. In an in vivo mouse model of focal epilepsy, these cuts are used to isolate the seizure‐prone cortex by severing connections in cortical layers II–IV, without injuring the overlying tissue and blood vessels, leading to dramatic reductions in seizure propagation.

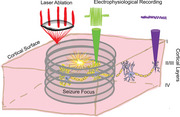

## 1 Introduction

One out of 26 people will develop epilepsy at some point in their lifetime and ≈50 million people worldwide are currently diagnosed with this disease.^[^ ^1^ ^]^ Epilepsy is also the most common neurologic disorder in dogs.^[^ ^2^ ^]^ Epilepsy is defined by chronic recurrent seizures, which are characterized by aberrant, rhythmic, synchronized brain activity that is frequently correlated with involuntary behaviors.^[^ ^1^ ^]^ Partial onset, or focal, epilepsy is a subclass of the disease characterized by seizure initiation from a consistent, localized region, followed by propagation of the seizure to surrounding brain tissue.^[^ ^3^ ^]^ Traumatic brain injury and stroke are the most common causes of focal epilepsy,^[^ ^4^ ^]^ but it can also have other etiologies, including cortical neoplasia or cortical dysplasia in children.^[^ ^4^, ^5^ ^]^ While medical management is preferred, this approach fails in ≈45% of focal epilepsy patients either due to initial or developed resistance to pharmacological seizure control.^[^ ^4^, ^5^, ^6^ ^]^ The most effective alternative therapy is to localize the epileptic focus using electroencephalogram (EEG) recordings and then remove the seizure focus through tissue resection, which risks leaving patients with neurologic or behavioral deficits.^[^ ^6^, ^7^, ^8^ ^]^ The most common location of epileptic foci is the temporal lobe, although partial onset epilepsy can also occur in the occipital, parietal, and frontal lobes.^[^ ^9^ ^]^ For such neocortical focal epilepsies only 32–67% of patients became seizure free after resection of the focus, and despite the reduction in seizures a significant percentage of these patients are left with various neurologic deficits including loss of vision, sensory loss, agnosia, dysgraphia or agraphia, acalculia, disturbances in body image, problems with higher order functions, and personality changes, which can be as detrimental to everyday life as the seizures the surgery intended to resolve.^[^ ^9^, ^10^ ^]^ In cases where the seizure focus is surgically inaccessible, a newer technique, laser interstitial thermal therapy (LITT), can be used, where an infrared, continuous‐wave laser (Nd:YAG) is delivered using optical fibers and catheters to thermally ablate the entire seizure focus, over a region often spanning several centimeters.^[^ ^11^ ^]^ Based on a recent metanalysis, LITT ablation of epileptic foci left between 45% and 75% of patients seizure free after surgery, but surgical complications emerged in ≈5% to 30% and included neurologic deficits like blindness, cranial nerve deficits or paralysis, chronic pain, stroke, hemorrhage, persistent seizures, memory loss, hormonal imbalances with damage to the hypothalamus (SIADH and hyperphagia), and death.^[^ ^12^ ^]^

Recent work has improved the understanding of the underlying physiology behind seizure initiation and propagation. In rodents, acute seizures (e.g., those initiated by focal injection of chemoconvulsants into the cortex) have been found to initiate in layer V of the neocortex, spread to overlying neurons in layers II/III, and then propagate away from the seizure focus through these supragranular layers.^[^ ^13^ ^]^ This mechanism has been shown to underlie seizure propagation in multiple animal models of acutely induced focal seizures ^[^ ^13^, ^14^ ^]^ and may facilitate the propagation of seizures in humans with focal epilepsy.^[^ ^13^, ^14^ ^]^ A less invasive surgical approach, multiple subpial transections, capitalizes on this concept of horizontal seizure propagation through the cortex and uses a metal, hook‐shaped instrument to make incisions in the cortex, beneath the surface vasculature, that isolates the seizure focus, with the goal of preventing seizure propagation.^[^ ^15^ ^]^ In this procedure, several cuts through the focus are made, by dragging the wire hook through the brain, which is difficult to control, leading to variable cut angles and significant tissue damage.^[^ ^16^ ^]^ Follow‐up studies found that about two‐thirds of patients were seizure‐free, but with highly variable outcomes between patients, and up to 30% of patients had significant behavioral and neurologic deficits.^[^ ^17^ ^]^ Although the technique holds great promise, the current execution is difficult to control and not standardized, which has led to it being mostly abandoned at many epilepsy centers. A technique that enabled smaller, sub‐surface cuts in the cortex, precisely targeted to specific cortical layers, and resulting in less collateral damage could be used to improve and extend the concept of the multiple subpial transection procedure.

Tightly focused, infrared wavelength, femtosecond‐duration laser pulses enable micrometer‐sized cuts to be produced below the surface of the brain without affecting the overlying tissue.^[^ ^18^ ^]^ We previously showed in rats that femtosecond laser cuts in layers II–IV encircling a chemically‐induced, acute seizure focus led to a complete blockage of seizure propagation in 35% of animals and a 36% reduction, on average, in propagation in the remaining animals. These cuts caused minimal damage to the adjacent cortex and preserved the response of neurons inside the encircling cut to a peripheral stimulus.^[^ ^19^ ^]^ However, it remains unknown what efficacy such cuts have in chronic focal seizure models that more closely approximate human partial onset epilepsy, whether these cuts continue to block seizures over time after the initial injury from the laser cut has resolved, and what functional effect the cuts have on behavior.

In this paper, we test the hypothesis that (in part due to limited regeneration in the central nervous system ^[^ ^20^ ^]^) encircling sub‐surface laser cuts will lead to long‐term reduction of seizure initiation and propagation, while the precision of the cuts will prevent significant impacts on normal cortical function. We microinjected iron chloride into layer V of the cortex to induce chronic, focal, neocortical epilepsy in mice and used long‐term electrophysiological recordings to compare seizure propagation with and without femtosecond laser cuts that spanned layers II–IV and encircled the seizure focus. The iron chloride model was selected for this study because it leads to chronic seizures from a defined brain region, and it models aspects of traumatic brain injury‐induced focal epilepsy.^[^ ^21^ ^]^ We found that laser ablation reduces seizure propagation by 95% while having almost no detectable effect on a skilled forelimb reaching/grasping task. These results suggest that the sub‐surface cortical cuts with minimal collateral damage to surrounding tissue produced by tightly focused femtosecond laser pulses could provide a new approach to treating medically refractory partial‐onset epilepsy in patients.

## 2 Result

### 2.1 Laser Cuts in Layers II‐IV of the Cortex Encircling an Epileptic Focus Reduced Seizure Propagation

**Figure 1:**
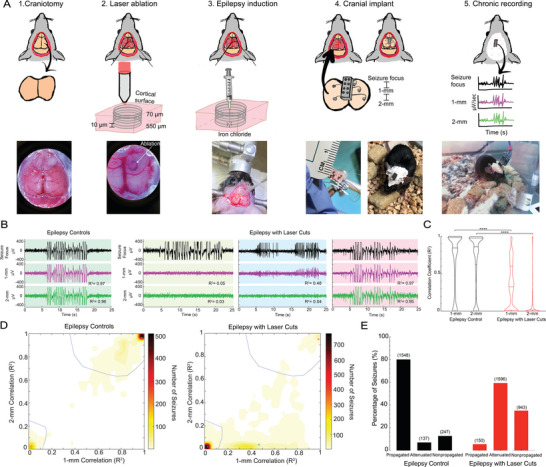
Laser microtransections encircling a chronic seizure focus significantly attenuated or blocked seizure propagation. A) Schematic representation of our surgical procedure. 1) Created a 4 × 10 mm^2^ craniotomy between lambda and bregma. 2). Laser ablation in 1‐mm diameter circles at every 10 µm from 550 µm (bottom of layer IV) to 70 µm (top of layer II) beneath the cortical surface. 3) Chronic focal seizures induced by microinjection of ≈350 nL of 100‐mm FeCl_3_ in saline into the center of the ablated cylinder. 4) Electrodes implanted in the bone flap at the seizure focus, 1‐mm away, and 2‐mm away. The bone flap was then reimplanted. 5) Animals were chronically recorded for 24 h once a week for 3, or more, months. B) Representative local field potential recordings with correlation coefficients, which indicate how well the recordings at 1‐mm and 2‐mm distances match the recordings from the seizure focus. Background colors are used to indicate where these seizures fall in the plots in C and D (indicated by the same color dots). C) Violin plots of all seizure correlation coefficients for epilepsy controls and epilepsy with laser cuts (red) (^****^ *p *< 0.0001, Kolmogorov–Smirnov test). D) 2‐D contour maps of the density of seizures as a function of the 1‐ and 2‐mm correlation coefficients, clustered into three groups termed: propagated (top right), attenuated (middle), and nonpropagated (bottom left). The representative seizures shown in B include: propagated in a control animal (left), then nonpropagated, attenuated, and propagated seizures in a laser‐cut animal (3 right panels). E) Bar graph indicating the percentage and number of seizures that fell into the three clusters (propagated, attenuated, or non‐propagated) for both the epilepsy control and epilepsy with laser cuts. The clustering was statistically different between laser cut and control groups (*p *< 0.0001, Chi‐squared test of associations).

To test the hypothesis that sub‐surface cortical laser cuts circumscribing a seizure focus can attenuate propagation, we produced cuts in craniotomized mice spanning from 550 to 70 µm beneath the cortical surface creating an open cylinder with a 1‐mm diameter (Movie S1, Supporting Information). These cuts were produced using tightly focused, femtosecond‐duration laser pulses, which, at sufficient energy, can produce micrometer‐scale tissue disruption at the laser focus due to the nonlinear absorption of laser energy, without causing significant collateral damage.^[^ ^18^ ^]^ We first optimized the cutting speed and depth‐dependent laser energy used to produce cuts with a uniform thickness of ≈55 µm over the 0.5‐mm depth range needed to cut layers II–IV (Figure S1, Supporting Information). In order to ensure that all connections in layer II/III were severed we cut from the bottom of layer I through layer IV. These cuts were 85% complete, on average, which was largely dependent on the presence of large surface blood vessels that attenuated the incident laser pulse and blocked ablation from occurring in some locations (Figure S2, Supporting Information). We used these optimized laser parameters — together with manual increases in laser power when focusing directly below a large blood vessel to try to increase the completeness of the cut — for our experiments testing the efficacy of circumscribing cuts for seizure attenuation. After laser cuts were placed in craniotomized mice, iron chloride (FeCl_3_) was microinjected at the center of the cylindrical cut, which led to focally initiated seizures within 2 weeks. We then placed three electrodes in the bone flap and reimplanted it so that one electrode was at the seizure focus and the other two were at distances of 1 and 2 mm from the seizure focus. Beginning ≈2 weeks after surgery, we recorded electrocorticography (ECoG) for 24 h once a week for 1–52 weeks (most mice 8–12 weeks) (Figure 1A). We identified epileptiform events as ECoG recordings from the seizure focus that were above a threshold and then manually characterized them as seizures (4621 events across all mice; e.g. Figure 1B), polyspikes (39 288 events; e.g. Figure S3A, Supporting Information), or interictal spikes (61 528 events; e.g., Figure S4A, Supporting Information), following standard criteria.^[^ ^22^ ^]^ We included animal groups with FeCl_3_ or saline injection and with or without laser cuts, named as follows: epilepsy with laser cuts, epilepsy control (no laser cuts), laser cut control (no epilepsy), and surgical control (no epilepsy and no laser cuts).

Interictal spikes and polyspikes were both regularly recorded in all groups (laser cut controls (95+/−42 & 24+/−35 events/day, respectively) and surgical controls (155+/−98 and 46+/−50)), but at increased incidence in animals with FeCl_3_ injection (epilepsy with laser cuts (600+/−486 and 363+/−517) and epilepsy controls (363+/−98 and 274+/−115)) (Figure S5, Supporting Information). Seizures were nearly absent in the laser cut control (0.3+/−0.6 seizures/day) and surgical control groups (0+/−0) but occurred at an incidence of ≈52+/−24 and 46+/−72 seizures/day in the epilepsy control and epilepsy with laser cut groups, respectively (Figure S5, Supporting Information). Epilepsy controls had a total of 1932 seizures (*n* = 4 mice) while epilepsy with laser cuts animals had 2689 total seizures (*n* = 7 mice) (Figure 1E).

We calculated the correlation coefficient between the ECoG recording at the seizure focus and the recordings taken at distances of 1 and 2 mm (Figure 1B). In the epilepsy controls, the electrophysiology recordings at 1 and 2 mm were highly correlated with the one at the seizure focus, while for epilepsy with laser cuts group, the correlation was reduced and more variable at 1 mm and was reduced even further at 2 mm, causing the distributions of the two groups to be significantly different from one another (*p* < 0.0001, Kolmogorov‐Smirnov test) (Figure 1C). Similar correlation analysis of the propagation of polyspikes (Figure S3B, Supporting Information) and interictal spikes (Figure S4B) showed significantly different correlation coefficients at 1 and 2 mm in epilepsy with laser‐cut animals, as compared to the epilepsy controls.

To characterize the propagation of individual epileptiform events, we separately examined seizures (Figure S6A, Supporting Information), polyspikes (Figure S6B, Supporting Information), and interictal spikes (Figure S6C, Supporting Information), combining data from all animals with FeCl_3_ injections. These events were clustered based on the correlation coefficients of the ECoG recordings at 1 and 2 mm, relative to the one at the seizure focus, using the Mahalanobis distance‐based clustering algorithm.^[^ ^23^ ^]^ We first varied the number of clusters and found that using three clusters reliably led to similar group boundaries, more clusters led to groupings that were inconsistent and sometimes nonsensical (e.g., dividing a dense mass of events). With three clusters, events grouped into a compact cluster with high correlation at 1 and 2 mm – a “propagated” event; another cluster with low correlation at 1 and 2 mm – a “nonpropagated” event; and into a lower density cluster that tended to have reduced correlation at 1 mm and further reduced correlation at 2 mm – an “attenuated” event (Figure S6, Supporting Information). The laser cuts produced a significant difference in the propagation of epileptic events (Figure 1D). With the clusters we defined, we found that with laser cuts only 5% of seizures propagated, 60% were attenuated, and 35% did not propagate, as compared to 80% propagated, 7% attenuated, and 13% non‐propagated without laser cuts (*p *< 0.0001, Chi‐squared test of association) (Figure 1E). We also found nearly as strong reductions in the propagation of polyspikes (Figure S3C,D, Supporting Information) and interictal spikes (Figure S4C,D, Supporting Information) with laser cuts.

### 2.2 Seizures Blocked by Laser Cuts Were as Powerful as Propagated Ones in Controls

**Figure 2:**
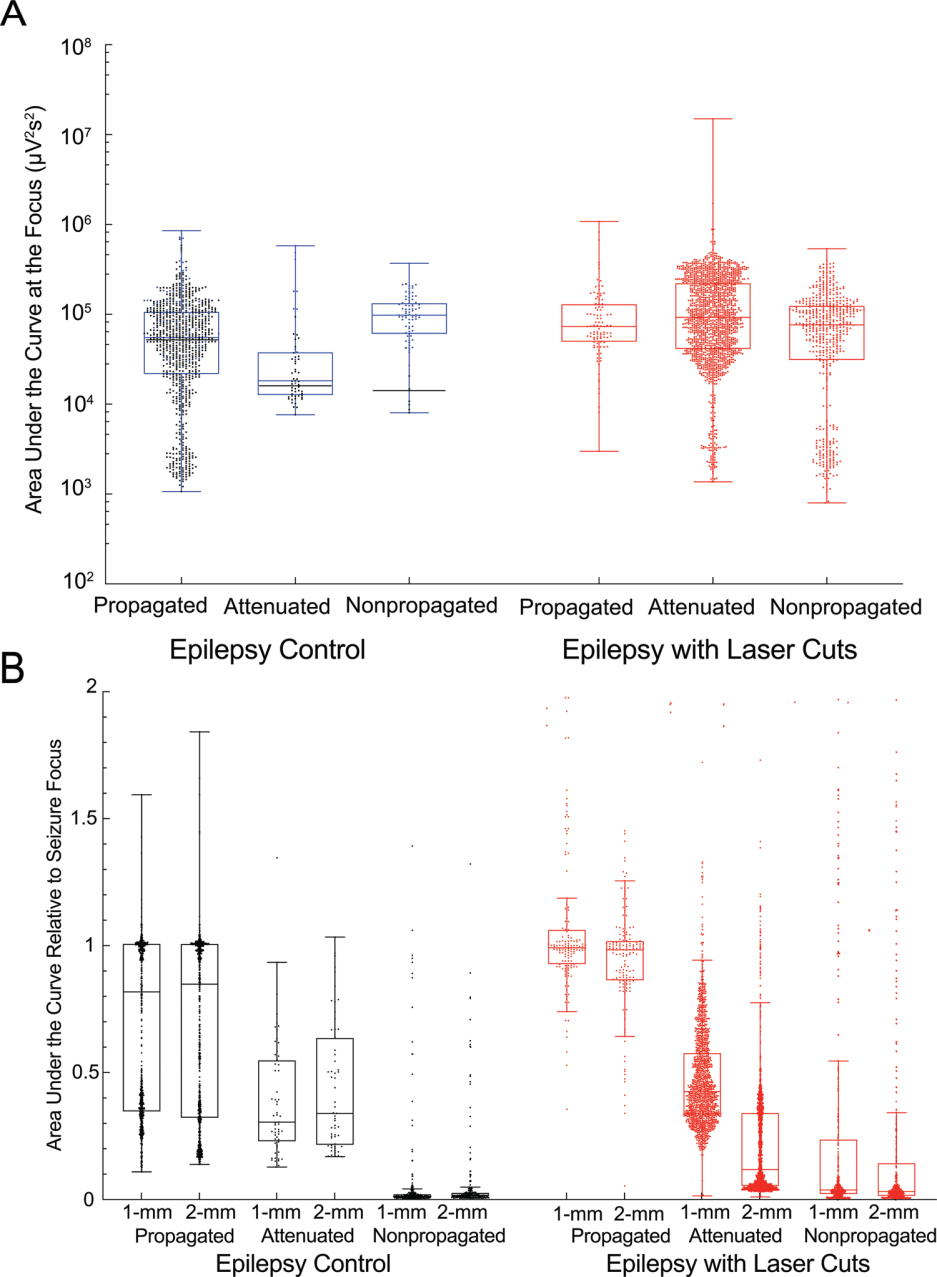
Nonpropagated and attenuated seizures in laser‐cut animals were as powerful as propagated seizures in epilepsy controls. A) Area under the curve for seizures with less than 10% of the points saturating at the focus, comparing the total power of seizures in each cluster between epilepsy with laser cuts (red) and epilepsy control (black/blue). Dots represent individual seizures (blue dots indicate an outlier animal). B) Area under the curve at distant electrodes, normalized to that at the seizure focus, comparing clusters (propagated, attenuated, and non‐propagated) between epilepsy control and epilepsy with laser cuts (red) (dots represent individual seizures).

To rule out the possibility that attenuated or non‐propagated seizures in mice with laser cuts were just less powerful at the seizure focus, we used the ECoG recordings from the seizure focus to calculate several seizure metrics, including maximum band power (over 0–50 Hz), seizure duration, and integrated band power over the seizure – termed the area under the curve (AUC). We had chosen to set the gain on the electrophysiological amplifiers so that powerful seizures were partially saturated, with the goal of minimizing the chance that weak, propagated seizures were difficult to pick out from the noise in our analysis of propagation probability. To reduce the influence of this saturation on the measurement of seizure metrics like AUC, we removed all events that had more than 10% of the data points saturated (28% of all seizures removed, of which 60% were from epilepsy control and 40% were from epilepsy with laser cuts). Removing saturated recordings enabled measurement of these metrics, but admittedly biases this analysis toward slightly weaker seizures. In control mice, attenuated seizures were significantly less powerful than propagated seizures, as intuitively expected (**Figure** **2**A). However, the non‐propagated seizures appeared to be equally as powerful as propagated ones. We found that this was dominated by a single control mouse with 89% of the nonpropagated seizures (blue data points and median indicators in Figure 2A). With this mouse excluded, both non‐propagated and attenuated seizures had lower AUC, max band power, and duration as compared to propagated seizures in control mice (Figure 2A, Figure S7A,B, Supporting Information; black median indicators; *p* = 0.0007, linear mixed effects model, Tukey post hoc multiple comparisons correction). In mice with laser cuts, we found no difference in the AUC or duration at the seizure focus between propagated, attenuated, and nonpropagated seizures (Figure 2A; Figure S7B, Supporting Information), however the max band power was slightly higher in propagated seizures, suggesting stronger seizures were more likely to propagate past the cuts (Figure S7A, Supporting Information). Critically, there was no difference in the power of propagated, attenuated, or nonpropagated seizures in mice with laser cuts when compared with propagated seizures in control mice, indicating that without the laser cuts these seizures would likely have propagated. We also characterized the time of day during the 24‐h recording period that seizures occurred and found no differences between laser cut and control groups or between propagated, attenuated, or nonpropagated seizures (Figure S7C, Supporting Information).

The duration, max band power, and AUC of seizures measured at the distant electrodes (and normalized to the measurements from the focus) were each decreased for attenuated and nonpropagated seizures, as compared to propagated seizures, for mice both with and without laser cuts (Figure 2B; Figure S8A,B, Supporting Information; *p *< 0.0001, linear mixed effects model, Tukey post hoc multiple comparisons correction). This suggests clustering seizures based on the correlation between focal and distant ECoG recordings distinguished electrophysiologically meaningful propagation, attenuation, and nonpropagation. Defining seizure arrival as the time when seizure power exceeded a threshold (average band power over the entire recording session plus one standard deviation), we measured the delay for seizures to propagate from the focus to distant electrodes and found no difference between mice with and without laser cuts (Figure S8C, Supporting Information).

### 2.3 Additional Encircling Cuts Did Not Improve Blocking Efficacy, But Revealed That Laser Cuts Reduced Seizure Initiation

**Figure 3:**
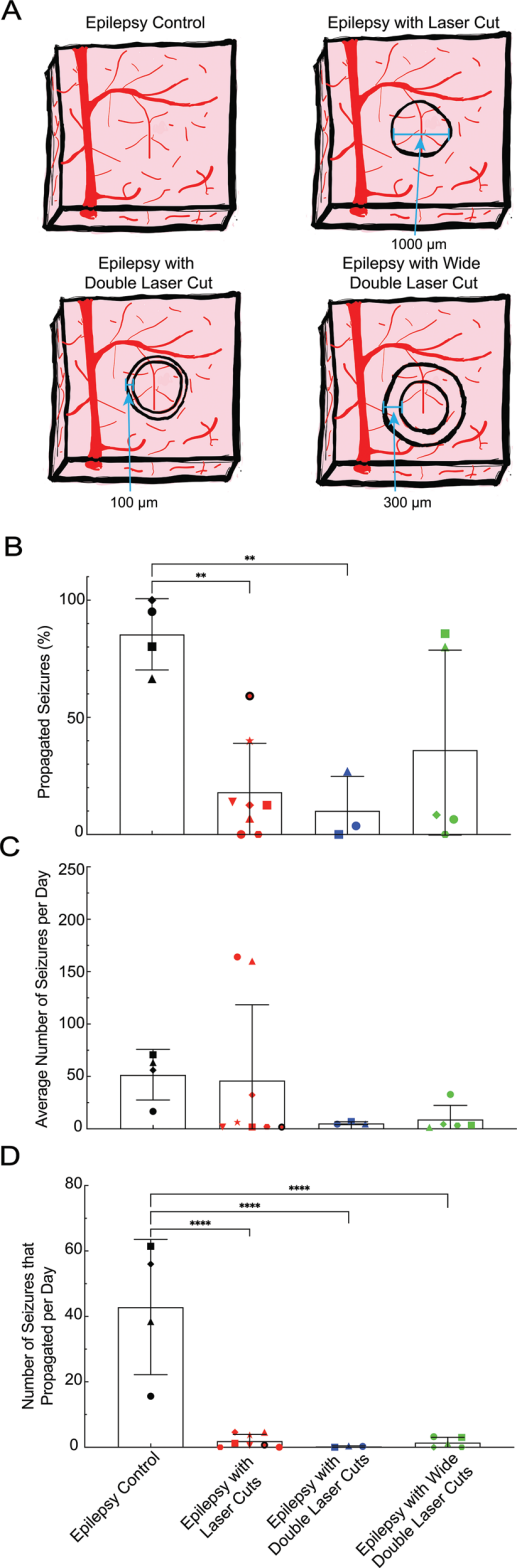
Closely‐ and widely‐spaced double‐cuts did not further decrease the percentage of propagated seizures but did decrease the number of daily seizures. A) Schematic representation of the different laser‐cut geometries. B) Percentage of seizures that propagated in epilepsy control, single‐cut, close double‐cut, and wide double‐cut animals (^**^ *p* = 0.004, one‐way ANOVA, Tukey post hoc multiple comparisons correction). C) Comparison of the average number of seizures per day between controls and the three types of laser cuts made. D) The average number of seizures per day that propagated in each group (^****^ *p *< 0.0001, one‐way ANOVA, Tukey post hoc multiple comparisons correction).

To test whether additional severing of lateral connections increases the percentage of seizures that are blocked, we placed close (100 µm) (Movie S2, Supporting Information) and widely (300 µm) (Movie S3, Supporting Information) spaced double‐cuts around the seizure focus (**Figure** **3**A). Neither closely nor widely spaced double‐cuts decreased the percentage of seizures that propagated (Figure 3B). We did find a trend toward a decreasing number of seizures initiated each day in animals with laser cuts: 52+/−24 in epilepsy controls, 46+/−72 in mice with single‐cuts (but with a bimodal distribution), 5+/−1 with closely‐spaced double‐cuts, and 3+/−1 with widely‐spaced double‐cuts (Figure 3C). In animals with single laser cuts, there was significant variability in the number of seizures that were initiated each day; nonetheless 63% of single‐cut and all but one of the double‐cut mice (87%) had fewer than seven seizures per day, an 87% reduction compared to controls. We did not see similar trends toward decreased numbers of interictal spikes or polyspikes with single‐cuts or either of the double‐cut geometries (Figure S9, Supporting Information). The decrease in seizure incidence together with the decrease in seizure propagation led to less than two seizures/day that propagated past the cuts, on average, for all cut geometries (Figure 3D). These results suggest that making extra cuts does not dramatically improve the attenuation of seizure propagation but may temper seizure initiation.

### 2.4 Seizure Blocking Efficacy Varied Across Mice, But Not Over Time

**Figure 4:**
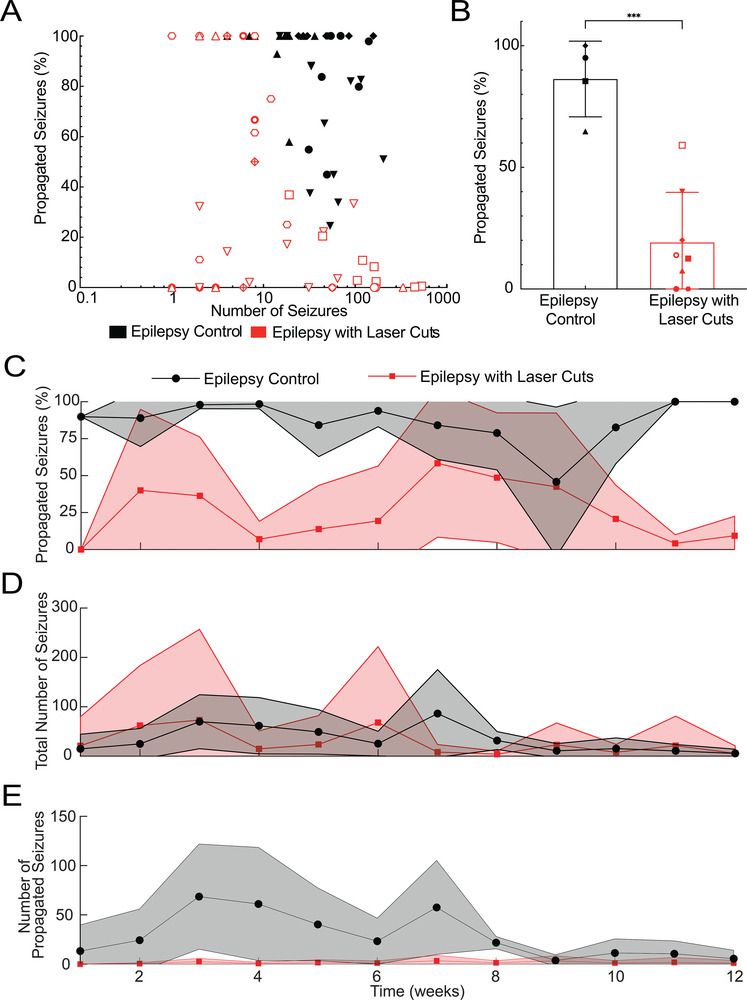
Laser ablation significantly reduced average seizure propagation across all animals and remained effective over time. A) Percentage of seizures that propagated as a function of seizure incidence for both epilepsy controls and epilepsy with laser cuts (red). Each animal is represented by a different shape, with recordings from multiple days for each animal. B) Average seizure propagation for each animal for epilepsy with laser cuts and the epilepsy control groups (^***^ *p* = 0.0001, unpaired t‐test). C) Percentage of propagated seizures, D) total number of seizures per day, and E) total number of propagated seizures per day, averaged across all animals, as a function of time for the epilepsy control group and the epilepsy with laser cut group (red).

The number of seizures and the fraction that propagated varied between recording sessions and between animals (**Figure** **4**A). Recording sessions from mice without laser cuts tended to have larger numbers of seizures and high propagation, while recording sessions from mice with laser cuts tended to have larger numbers of seizures with reduced propagation, or reduced numbers of seizures and highly variable propagation (Figure 4A). Averaging across all recording sessions for each mouse, we found that laser cuts reduced seizure propagation from 85.5% in controls (range: 66–100%) to 18% in treated mice (range: 0–59%) (*p* = 0.0002, *t*‐test; Figure 4B). Averaging across all mice, we found that the decreased propagation in laser‐cut mice did not significantly change out to 12 weeks (linear mixed model; Figure 4C). The number of seizures per day trended lower over time and was similarly variable in mice with and without laser cuts (Figure 4D), but due to reduced propagation, laser‐cut mice had, on average, less than two propagated seizures per day, as compared to 43 per day in control mice (*p *< 0.0001, *t*‐test, Figure 4E).

**Figure 5:**
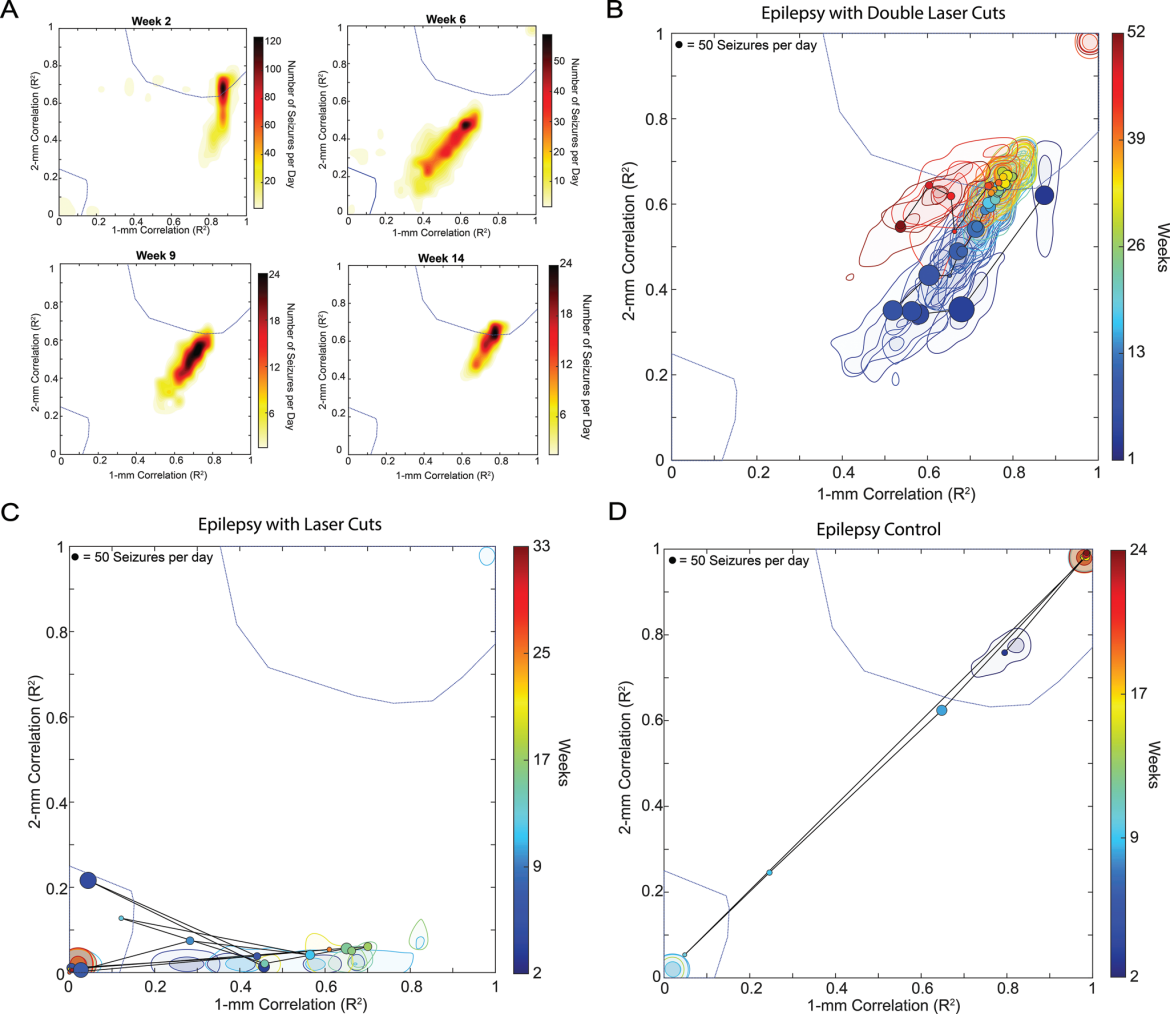
Seizure propagation blocking by laser cuts was stable over time. A closely‐spaced double‐cut, a single‐cut, and a control animal, each with a high incidence of seizures, were followed for an extended period of time to inspect the week‐to‐week changes in the efficiency of laser cuts in blocking seizures. A) 2‐D contour plots of the correlations of recordings of seizures at 1 and 2 mm with the recording from the seizure focus at different times for a mouse with closely‐spaced double‐cuts. B–D) 2‐D contour plots of correlation coefficient over time showing the average centroid and the 80th and 33rd percentile contours for the peak density of seizures for each week for mice with a closely‐spaced double‐ cut B), a single cut C), and no laser cuts D). The lines connect successive geometric medians of seizure density, with the color of the centroid indicating the recording time and the size of the centroid indicating how many seizures occurred during the 24‐h recording that week (scale in the top left corner). To have sufficient data to determine the averages of the correlation coefficients, we excluded data from any recording days that had fewer than ten seizures.

A few mice had greatly increased numbers of seizures, enabling us to define the efficacy of the laser cuts week‐by‐week for each of these mice over an extended duration. **Figure** **5** shows seizure propagation data from a mouse with closely‐spaced double‐cuts (Figure 5A,B), one with a single cut (Figure 5C), and a control (Figure 5D). In the double‐cut animal (which had so many seizures it was excluded from the quantitative analysis above to avoid bias), the seizure‐blocking efficiency improved over several weeks, then regressed back toward the efficacy shortly after the cuts were made where it stabilized from 18 to 52 weeks. For the single‐cut animal, blocking was highly effective and remained stable over ≈33 weeks. In the control animal, seizure propagation was high and stable over 24 weeks. Interestingly, both the double‐ and single‐cut mice showed a decreasing number of seizures per day over time, while seizure incidence was unchanged in the control animal.

### 2.5 Laser Cuts Cause Modest, Short‐Lived Blood Flow Decreases and Minor Chronic Tissue Changes

**Figure 6:**
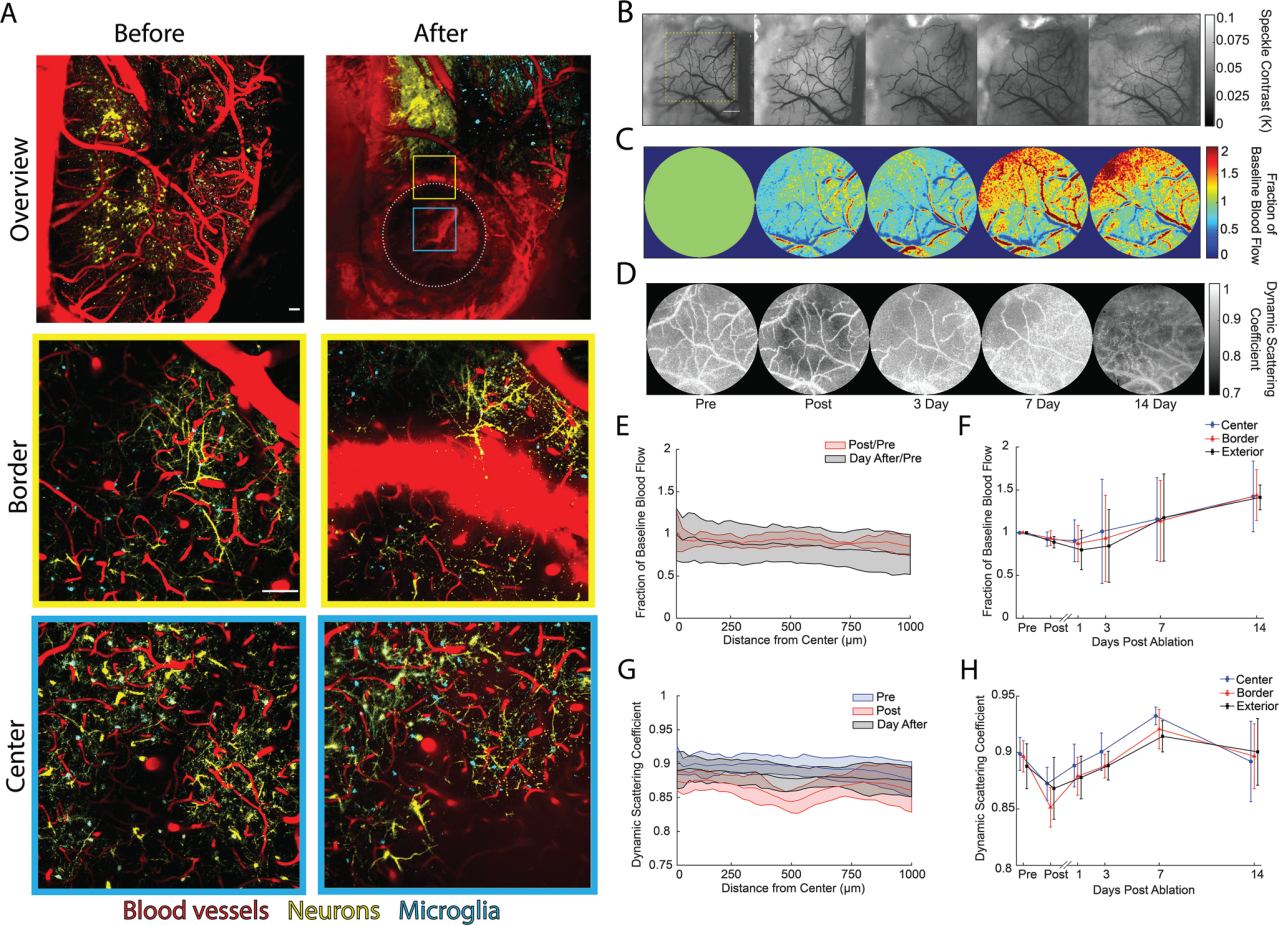
Laser cuts cause minimal collateral damage and only a modest, transient impact on local cortical blood flow. A) Two‐photon images showing blood vessels (red), neurons (yellow), and microglia (cyan). Top panel boxes show low magnification images taken before and after producing a 1‐mm diameter ablation from 550 to 70 µm beneath the surface of the cortex (white dotted circle: ablation, blue box: center, yellow box: border) (scale bar: 100 µm). The middle and bottom panels show zoomed‐in views of the border and ablated regions, respectively, before and after ablation (scale bar: 100 µm). B) Multi‐exposure speckle contrast images captured before, after, and 3, 7, and 14 days after ablation. Images show a single exposure time (25 ms). The ablated region is evident as a faint white ring in the post image (scale bar: 500 µm). Yellow dashed box indicates the field of view for images in panels C and D. C) Heat map showing the fractional change in blood flow over the 2 weeks recorded. D) Map of the fraction of light scattered by moving scatterers, such as flowing blood cells, over time. The cellular debris from the ablation shows up as a dark ring in the post image. E) Parenchymal blood flow, expressed as a fraction of baseline, as a function of distance from the cut center and measured immediately after and one day after the ablation. F) Fractional changes in baseline parenchymal blood flow for each region (inside the cut, at the border, and outside the cut) as a function of time. G) Fraction of light dynamically scattered as a function of distance from the cut center measured before, immediately after, and the day after ablation. H) Fraction of light dynamically scattered for each region as a function of time.

We used three approaches to characterize the physiological and tissue changes that result from the femtosecond laser cuts. First, we took multiphoton images of the tissue before and after producing cuts in mice expressing markers for microglia (CX3CR1‐GFP) and neurons (Thy1‐YFP) (**Figure** **6**A). Neurons and blood vessels remained intact at the center of the cut region. Extravasation of blood plasma into the tissue was apparent at the cut border, but nearby blood vessels and neurites remained intact (Movie S1, Supporting Information). No blood vessels on the brain surface were disrupted. Tissue was only ablated right at the cut border, where femtosecond laser pulses were focused causing multiphoton and avalanche ionization which vaporized tissue ^[^ ^18a^ ^]^ and led to a small cavitation bubble that displaced tissue.^[^ ^24^ ^]^ In the center of the cylindrical cut, microglia and neurite morphologies remained unchanged over hours after the ablation, suggesting minimal acute impacts at this distance from the cut. Overall, there were minimal changes found in vivo after laser ablation in regions even tens of micrometers away from the cut locations.

Second, we used multi‐exposure speckle imaging (MESI) to assess the impact of laser ablation on brain tissue perfusion over 2 weeks after laser ablation in these same mice (Figure 6B). MESI enables the cortical perfusion (Figure 6C) and the fraction of optical scatterers that are moving (Figure 6D) to be extracted from fits to the exposure‐time‐dependent speckle contrast.^[^ ^25^ ^]^ We first analyzed the impact shortly after the cut as a function of radial distance from the cut center and found only a weak trend toward decreased tissue perfusion with no clear dependence on distance from the cut (Figure 6E). There was a clear decrease in the fraction of light scattered by moving tissue components ≈500 µm from the cut center, at the location of the cut (Figure 6F). Based on this finding, we examined how perfusion and the degree of dynamic scattering changed over time in regions radially outward relative to the cut center defined as: inside the cut (≤450 µm from the center), at the border (450 µm < d ≤ 550 µm), and outside the cut (550 µm < d ≤ 1000 µm). There was a minor blood flow decrease after the cut that recovered to baseline within 3 days and then increased above baseline flow through the 2 weeks of observation (Figure 6G). This increase in flow did not vary strongly across the three regions and was associated with vascular remodeling at the brain surface (evident in speckle contrast images at later times; Figure 6B). The fraction of dynamically scattered light decreased immediately after the laser cuts, most strongly at the cut border, likely associated with the bleeding and extravasation of red blood cells into the brain tissue at the cut and onto the brain surface (Figure 6H). The fraction of dynamically scattered light returned to normal within a few days.

**Figure 7:**
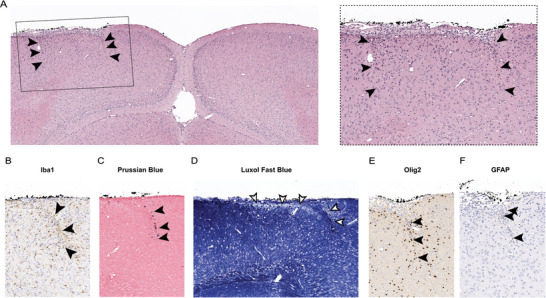
Thirty days after laser cuts the cortical structure was intact and had minute scarring with inflammatory infiltrate, minor gliosis, and mild loss of myelin. A) The left panel shows a light microscope picture of a 5‐µm thick coronal section through the cylindrical laser cut, stained with hematoxylin and eosin (H&E), with arrowheads indicating the top, middle, and bottom of the cuts. The dashed box indicates the location of the panel on the right showing a zoomed‐in image of the laser cuts. Additional panels show histological stains and immunohistological labeling at the location of laser cuts for: B) Iba1 (microglia/macrophages), C) Prussian blue (hemosiderin), D) Luxol fast blue (myelin), E) Olig2 (oligodendrocytes), and F) glial fibrillary acid protein (GFAP; astrocytes) (scale bars: 100 µm).

Third, we performed histology 1 month after laser cuts were made to determine the chronic effect of laser cuts on tissue architecture. Coronal sections of the brain, including the area of the laser cut, were examined using histochemical stains and immunohistochemistry. The area of the cut was marked on the cortical surface using surgical ink, and the contralateral cortex was used as a control.  Hematoxylin and eosin (H & E) staining revealed linear areas of hypercellularity along the trajectory of the laser cut and in the adjacent leptomeninges, as well as mild disorganization of the neuronal layers in the cortex between the cuts, but no notable disruption of cortical structure (**Figure** **7**A). Tissue fixation has been shown to shrink sections by 25%,^[^ ^26^ ^]^ therefore the diameter of the ablated cylinder was found to have shrunk from 1 to 0.8 mm and the height of the cut border through layers II–IV shrank from 0.5 to 0.4 mm. Despite the shrinkage of tissue, it is still clear when inspecting the cellular organization of the cortex that laser cuts severed layers II/III and cut into the top of layer IV. Acute histology to optimize laser cuts indicated that cuts were 55 µm in thickness immediately after ablation (Figure S2, Supporting Information). Histology 1 month later showed that cuts left a scar with, on average, 20‐µm thickness. At the cut, there was an increase in the density of cells that were immunoreactive for Iba1, a marker of brain microglia and, potentially, invading macrophages (Figure 7B). Prussian blue staining for iron further revealed intracytoplasmic accumulation of hemosiderin in most of these Iba1‐positive cells (Figure 7C). Luxol fast blue staining for myelin showed mild myelin loss superficially inside the ablated cylinder (Figure 7D). Immunohistochemistry for olig2 (oligodendrocyte marker) (Figure 7E) and glial fibrillary acidic protein (astrocyte marker) (Figure 7F) showed a very mild increase in these glial cells along the cuts. Cellularity and tissue morphology inside the cuts were similar to the contralateral cortex (Figure 7A).

### 2.6 Encircling Laser Cuts in Motor Cortex Did Not Cause Chronic Deficits in a Complex Reaching Task

**Figure 8:**
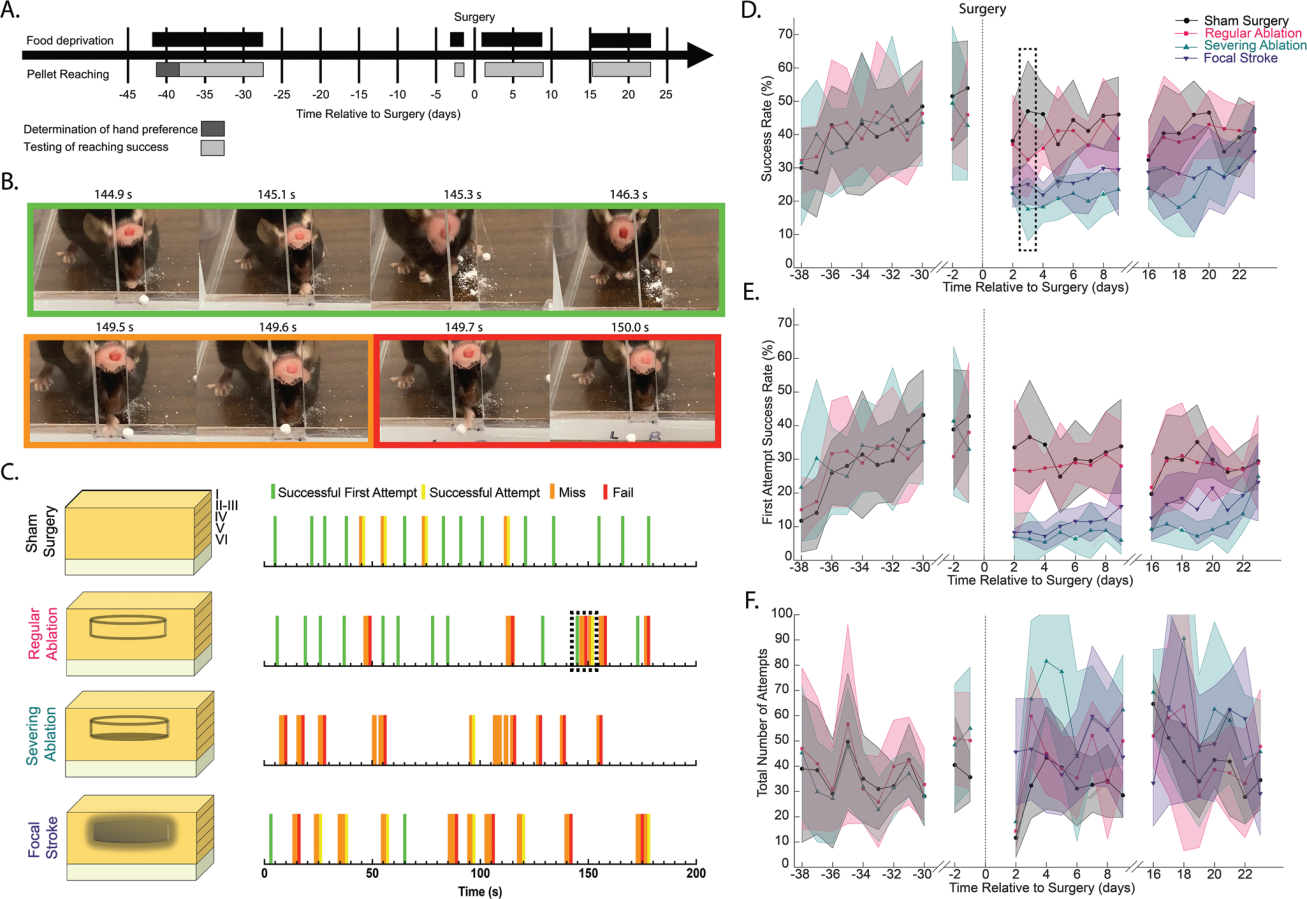
Regular encircling laser ablation targeted to the forepaw motor cortex did not reduce long‐term ability to perform skilled reaching tasks. A) Timeline of when food deprivation and pellet reaching training and testing occurred during the pellet reaching experiment. B) Video snapshots of a first‐time successful attempt where an animal grabs and eats the pellet on the first try (green), a miss where an animal reaches and cannot grab the pellet (orange), and a fail where the animal knocks the pellet out of reach (red). C) Schematic representation of a cortical column with the surgical treatment for each of the four experimental groups: sham, regular ablation, a “severing ablation” where the bottom of the cylinder is also laser cut, and a focal photothrombotic stroke. To the right, the panel shows graphical representations of the success of a representative animal's reach for a sucrose pellet in the first 3 min of their recording on day 3 postsurgery, for each group. The dashed line box in the regular ablation row indicates the reaches that are shown in panel B. D) Average success rate, E) average first attempt success rate, and F) total number of attempts to grab and eat a pellet during training and after surgery for all four groups (*n* = 6–7 mice/group). The dashed line box in (D) indicates the timepoint of the representative data shown in (C).

To test for any acute or long‐lasting behavioral deficits caused by the laser cuts, we placed cuts in the caudal forelimb area of the motor cortex (Figure S10, Supporting Information) and studied performance in a skilled forelimb pellet reaching task (**Figure** **8**A,B). We compared mice with a regular ablation to mice with sham surgeries and mice with focal photothrombotic strokes, which were expected to show a deficit (Figure 8C).^[^ ^27^ ^]^ To assess the impact of a more extensive laser ablation that also severed vertical cortical connections (and approached the invasiveness of resection), we included a group of mice with the same cylindrical laser cut, but with the bottom of the cylinder also severed by laser ablation (Figure 8C) (Movie S4, Supporting Information). We found a significant decrease in the pellet retrieval success (Figure 8D) and first attempt pellet retrieval success (Figure 8E) in mice with focal strokes (26% overall success, 11% first attempt success; averaged over the first week) and with severing ablations (21%, 7%), as compared to the sham group (43%, 32%; *p *< 0.0001 for overall success and first attempt success of focal stroke and severing ablation vs sham, one‐way ANOVA with Tukey's multiple comparisons test). This deficit did not show any improvement until 3 weeks after the surgery, indicating that laser ablation can cause a deficit and that deficit comes from severing both vertical and horizontal connections. In contrast, mice with the standard laser cuts showed only a very modest decrease in pellet retrieval success (39%, 28%; *p* = 0.03 for the overall success of regular ablation vs sham) in the early days after surgery (Figure 8C,D) and had performance matching sham mice at all other time points. There was little variation in the pellet reaching attempts across all groups and time points, except for mice with severing ablations, which made modestly more attempts in the early days after the surgery (Figure 8F). Histology at 1 month showed that severing ablations led to a collapse of tissue structure at the cortical surface (Figure S11A, Supporting Information), accompanied by hypercellularity within the ablated region comprised of macrophages and microglia (Figure S11B, Supporting Information), hemosiderin deposition (Figure S11C, Supporting Information), loss of myelination at the cortical surface (Figure S11D, Supporting Information), and increases in both oligodendrocytes (Figure S11E, Supporting Information) and astrocytes (Figure S11F, Supporting Information). In conclusion, the laser ablation pattern that effectively blocked seizure propagation did not notably impact performance on a complex forelimb motor task when placed in the relevant cortical region, while the severing ablation had as severe a behavioral impact as a focal stroke. In conjunction with this, adding just a 10‐µm thick cut that severed vertical connections at the bottom of the cylindrical cut led to more severe tissue impacts 1 month after ablation, with cylindrical cuts leaving the cortex almost indistinguishable from the contralateral side (Figure 7) while after severing ablations the entire encircled region appeared to be a hypercellular glial scar (Figure S11, Supporting Information).

## 3 Discussion

Sub‐surface cuts produced by tightly‐focused infrared femtosecond laser pulses localized to cortical layers II–IV and encircling a seizure focus were able to block horizontal propagation of focal seizures with high efficacy while minimally affecting normal brain structure or the ability to perform a complex reaching task. With over 100 000 epileptiform events measured in this study, we found laser cuts reduced the propagation of interictal spikes, polyspikes, and especially seizures, for which propagation was reduced from 80% in control animals to 5% in laser‐cut animals. Seizures that do not propagate, if restricted to a small enough area, may effectively be similar, clinically, to seizures that never occurred. Moreover, in combination with medication, such cuts might be even more efficacious. Epilepsy with laser‐cut animals had a higher number of polyspikes (9%) and interictal spikes (26%) propagate compared to seizures, but the propagation of these events was still significantly reduced when compared with epilepsy controls (67% propagated for both polyspikes and interictal spikes). We hypothesize that these smaller electrical events propagated due to a lower threshold of neuron activation needed to maintain their propagation and therefore minor breaks in the cut border may have provided sufficient intact neurites for them to propagate through to distant electrodes.^[^ ^28^ ^]^ After removing seizures with substantial saturation, we found there were no differences in seizure power, duration, or other metrics, as measured at the focus, between attenuated and nonpropagated seizures in mice with laser cuts and propagated seizures in control mice, indicating that blocked seizures likely would have propagated without the laser cuts. To try and improve the efficacy of this neurosurgical approach we made both close‐ and widely‐spaced double‐cuts, but without further notable decreases in seizure propagation. Cuts also tended to reduce the incidence of seizures, with ≈75% of all laser‐cut animals (including both single and double cut) showing an 87% reduction in the average number of daily seizures. This reduction in seizure initiation appeared more robust in animals with double cuts, while in mice with single cuts, there was a bimodal distribution, showing reduced seizure incidence in most animals and high seizure incidence in two mice, possibly reflecting the variability in seizure incidence previously reported with this model.^[^ ^21^ ^]^ Laser cuts also reduced propagated seizures on average by ≈96% for all cut geometries when compared with controls (from 41.6 to 1.4 propagated seizures per day). This reduction in seizure propagation was retained over 3 months to a year — consistent with the poor regenerative nature of the adult central nervous system ^[^ ^20^ ^]^ and mild glial scarring at the cut, which also may create a barrier to seizure propagation — suggesting that longer‐term efficacy may be possible (and which we demonstrated in a few animals that were followed longer). In earlier work, we showed that similar sub‐surface laser cuts around an acute seizure focus induced by intracortical injection of a chemoconvulsant reduced both seizure incidence and propagation.^[^ ^19^, ^29^ ^]^ In comparison, previous studies of the efficacy of the multiple subpial transection procedure in animal models suggested that while the cuts did effectively block kainic‐acid‐induced seizures ^[^ ^29^ ^]^ and decreased after‐discharges in response to cortical overstimulation,^[^ ^15^, ^30^ ^]^ they led to significant neurological deficits.

While laser ablation did not completely abolish seizure initiation or propagation, the reduction in seizure propagation achieved through laser cuts may have clinical implications. Seizure propagation plays a pivotal role in exacerbating symptoms through what is commonly referred to as a Jacksonian March, where symptoms spread and intensify across the body as the seizure propagates through the brain.^[^ ^3^, ^6^, ^31^ ^]^ By interrupting the spread of seizures, our approach thus holds promise for alleviating symptom progression and severity, potentially improving the quality of life for individuals with epilepsy.

Unwanted effects on tissue and brain function from these cuts are reduced by the fine cut width, minimal collateral damage, ability to produce sub‐surface tissue cuts, and the precise control of the cut location that ablation with tightly‐focused femtosecond laser pulses affords.^[^ ^32^ ^]^ In our previous work, femtosecond laser cuts were between 80 and 180 µm in width,^[^ ^19^ ^]^ which we reduced to ≈55 µm immediately after ablation and ≈20 µm after a month of healing while also increasing the uniformity and completeness of the cut in this work. These cuts did not injure any blood vessels on the surface of the brain and were completely localized inside the cortex, at the targeted cortical layers. They led to modest decreases in cortical blood flow that recovered in days, consistent with the sub‐surface nature of the cuts that completely preserved the vasculature on the brain surface. Minor tissue scarring and increased inflammatory cell density were evident at the laser cuts at 1 month, suggesting the laser cuts do not induce severe chronic pathology. Despite the bleeding from the laser ablation, animals with laser cuts but no FeCl_3_ microinjection did not develop epilepsy. The high precision and negligible collateral damage achieved with femtosecond laser ablation is why this tool has been adapted for surgical procedures, including ocular refractive surgery,^[^ ^32^, ^33^ ^]^ dental cavity removal,^[^ ^32^, ^34^ ^]^ and removal of bone fragments in orthopedic surgery (under development).^[^ ^35^ ^]^ We tested the effect of the laser cuts on motor function and found that there were only minor acute deficits in a complex reaching task, which quickly recovered. In contrast, severing vertical connections in conjunction with horizontal connections reduced motor function significantly – comparable to the impact of a focal stroke – and these animals did not quickly recover. These more severe laser cuts are likely closer to the surgical removal of the seizure focus often used clinically, which can lead to significant neurologic deficits.^[^ ^9^ ^]^ Previously, we showed that the somatosensory cortex encircled by similar laser cuts still responded to a peripheral stimulus, further supporting the notion of reduced neurological disruption from these cuts.^[^ ^19^ ^]^ Thus, the micrometer‐sized sub‐surface laser cuts that were able to block seizure propagation produced negligible structural damage and nearly absent functional impairment.

Previous work using acute seizure models has shown that focal seizures initiate in the pyramidal cells of layer V of the cortex and then propagate laterally through layers II/III.^[^ ^13^, ^14^, ^36^ ^]^ In cortical slices, only cells in layer V were able to initiate the synchronized activity that leads to a seizure,^[^ ^36a^ ^]^ and only layers II/III were capable of propagating this activity horizontally through the slice.^[^ ^36b^ ^]^ In both awake and anesthetized mice, seizures induced in layer V of the cortex relied on layers II/III connections for horizontal propagation.^[^ ^13^, ^14^ ^]^ This previous work examining the role of different cortical layers in focal seizure propagation relied on acute seizure models, such as microinjection of 4‐aminopyridine. These models reliably generate powerful seizures but fail to capture the long‐term changes in intrinsic neural properties, neural connectivity, or other aspects of the tissue microenvironment that occur during the formation of an epileptic focus.^[^ ^31n^ ^]^ Such processes could alter the role of different cortical layers in supporting seizure initiation and propagation. The iron chloride model used in this study creates a chronic seizure focus that develops over weeks and models aspects of what occurs after a traumatic brain injury.^[^ ^21^ ^]^ The significant reduction in the propagation of iron chloride‐induced seizures we found after severing layer II–IV connections is consistent with the idea that seizures in chronic epilepsy also propagate in a layer‐specific fashion.

About 5% of all epilepsy cases are due to traumatic brain injury (TBI) associated with military service, sports, accidents, or other inciting factors.^[^ ^37^ ^]^ A variety of effects secondary to TBI have been proposed to contribute to the formation of a seizure focus, including neuroinflammation,^[^ ^38^ ^]^ blood‐brain barrier permeability,^[^ ^39^ ^]^ tissue hypoxia,^[^ ^40^ ^]^ changes in neural excitability,^[^ ^41^ ^]^ reactive oxygen mediated damage from cerebral bleeding,^[^ ^42^ ^]^ and metabolic dysfunction.^[^ ^31^, ^43^ ^]^ The iron chloride microinjection model partially captures the development of a TBI‐induced seizure focus by simulating the accumulation of iron from the breakdown of hemoglobin left in the tissue after small microhemorrhages from a TBI.^[^ ^21^, ^31^, ^44^ ^]^ The iron accumulation is thought to lead to reactive oxygen species mediated membrane lipid peroxidation preferentially on glial cells, which reduces the removal of glutamate from the synaptic cleft, leading to overall hyperexcitability and the eventual development of a seizure focus.^[^ ^21^, ^31^, ^44^ ^]^ While no model perfectly captures human focal epilepsy, we chose the iron chloride model for quick development and regular seizure activity. Other animal models of focal TBI, including the fluid percussion injury and controlled cortical impact models, also lead to focal seizures, but these more complex models normally have fewer than ≈1 seizure/day. Pediatric cases of medically refractory focal epilepsy are often associated with cortical dysplasia, which can be modeled in mice using in‐utero electroporation to constitutively activate mTOR in cortical pyramidal neurons in a focal cortical region.^[^ ^45^ ^]^ Evaluating our procedure in these models would require localization and mapping of the seizure focus or foci, which varies in location, size, and shape in these genetic models, and then planning and executing a mouse‐specific laser cut geometry. Future work should investigate the capability of encircling laser cuts to attenuate the seizures that are produced by these injuries and genetic models of focal epilepsy.

One limitation of the current study is that cuts were placed at the time of induction of epileptogenesis, which could impact the formation of the seizure focus. This was necessary due to the significant variability in seizure initiation with this model,^[^ ^21^ ^]^ which required us to record from epilepsy initiation so we could exclude animals that did not develop seizures, and the need for an open craniotomy for laser ablation but also skull‐fixed electrodes for long‐term electrophysiological recordings at the seizure focus and distant locations. However, we found that seizures were equally powerful, and had similar max band power and duration, between animals with laser cuts and those without, showing that a seizure focus did develop even with laser cuts. This finding also indicates that the decreased propagation we observed with laser cuts is not due to weaker seizures. There was a trend toward decreased seizure frequency in animals with laser cuts. This could be explained as an influence of the cuts on the progressive development of the epileptic focus. However, it is also possible that the reduced seizure frequency was due to the isolation of the seizure‐prone cortex from its adjacent tissue. Previous research suggests a minimum contiguous cortical area that can sustain synchronous activity is necessary for seizure initiation.^[^ ^3^, ^13^, ^46^ ^]^ By disrupting lateral cortical connections our cuts may decrease the area of the epileptic cortex that can easily synchronize, potentially making seizure initiation more difficult.

One remaining question is how to translate this approach into the operating room. With the laser system used in this study, we have demonstrated cortical transections as deep as ≈1 mm, but this will be inadequate in larger and more complex brains.^[^ ^18^ ^]^ The human cortex is not only thicker (1–4.5 mm) but is also convoluted with gyri and sulci.^[^ ^47^ ^]^ This presents three problems: first, the need for increased light penetration to achieve laser ablation at greater depths; second, the need for a laser delivery system that can traverse the folds of the brain without damaging the surrounding tissue; and third, the need to localize the seizure focus and determine the correct depths of layer II/III in that region. With longer wavelength laser light, tissue scattering is reduced while linear absorption by water and lipids increases, leading to optimal wavelengths for tissue penetration at the 1.3 and 1.7‐µm local minima in water absorption.^[^ ^48^ ^]^ At these wavelengths, targeted tissue cuts as deep as 5 mm (1.3‐µm pulses) and ≈1 cm (1.7‐µm pulses) are theoretically possible.^[^ ^18^ ^]^ Using these wavelengths is also likely to decrease the impact of large surface blood vessels on sub‐surface ablation, as they did for 2PEF imaging.^[^ ^49^ ^]^ Laser technologies that could produce energetic, femtosecond pulses at these wavelengths are becoming increasingly mature (high‐energy Yb femtosecond laser driving an optical parametric amplifier ^[^ ^50^ ^]^ or other nonlinear conversion process ^[^ ^51^ ^]^). To enable cortical cuts within the folds of the cortex, a probe that gently penetrates sulci and “side fires” into the cortex could be used. Supporting such a possibility, a flexible, sulci‐penetrating needle probe that can enable multiple cortical regions, both on top of gyri and inside sulci, to be reached from a single skull burr hole has recently been demonstrated in cadavers.^[^ ^52^ ^]^ Researchers were able to penetrate and readily transverse the sulci and gyri of cadavers, an approach that may find broad use in vivo for the delivery of treatments for tumors, strokes, and other neurodegenerative disorders.^[^ ^52^ ^]^ Lastly, determining the location and layer‐dependent depth of the seizure focus could pose a challenge for human applications of laser ablation. The mouse neocortex is relatively uniform which allowed us to roughly estimate the layers of the neocortex despite minor deviations in the location of the laser cuts in each animal. This contrasts with the human cortex with its convolutions and variations in cortical thickness that will play a larger role in determining the trajectory of our laser ablation. The locations of seizure foci can be mapped with millimeter precision utilizing intracranial ECoG, which is currently standard for the planning of surgical resections of epileptic foci.^[^ ^53^ ^]^ Newer MRI imaging techniques, including myelin mapping, laminar, and diffusion MRI, can determine cortical thickness non‐invasively, while ultrasound and optical coherence tomography could provide intraoperative assessments of cortical layer thickness.^[^ ^46^, ^54^, ^55^ ^]^ With these approaches, localization of the seizure focus and determining the appropriate depths needed for laser ablation to target the right cortical layers should be feasible in higher‐order mammalian brains such as humans or dogs. With longer wavelength femtosecond pulses, a sulci‐penetrating probe that can focus the light into the tissue, and anatomically‐registered robotic guidance of probe motion, our procedure could provide a new neurosurgical approach to treating cortical focal epilepsy in patients.

## 4 Experimental Section

### Study Design

Detailed descriptions of relevant materials and methods are in the Supporting Information. All animal experiments were approved by Cornell's Institutional Animal Care and Use Committee. The aim of this study was to determine the effect of encircling micrometer‐wide laser cuts that severed lateral connections in layers II–IV of the neocortex on the initiation and propagation of seizures from an induced epileptic focus, as well as to determine the impact of such cuts on the structure and function of surrounding brain tissue. To induce an epileptic focus, iron chloride was microinjected into the cortex of craniotomized mice. Tightly focused femtosecond laser pulses were then used to produce sub‐surface cuts encircling the injection site. Using implanted intracortical electrodes, electrophysiological ECoG recordings were measured from the seizure focus and at electrodes placed 1 and 2 mm away for 12–52 weeks and seizure propagation was quantified by examining the correlation of the distant recordings with the seizure focus. Four groups were examined: epilepsy controls (*n* = 4) (induced with seizures but did not have laser cuts), epilepsy with laser cuts (*n* = 8) (induced with seizures and had laser cuts), laser cut controls (*n* = 3) (given a sham intracortical injection, but had laser cuts), and surgical controls (*n* = 3) (given a sham intracortical injection and had no laser cuts). The effect of adding concentric laser cuts with “close” (*n* = 4) and “wide” (*n* = 5) spacing in mice with epilepsy was further tested. To assess the effects of laser cuts on the structure and function of the cortex, several approaches were used. Mice were imaged (*n* = 5) with fluorescently labeled neurons and microglia using multiphoton microscopy before and after ablation to see the acute effect on tissue architecture. Cortical blood flow changes were followed in the same mice over 2 weeks after ablation using multi‐exposure laser speckle imaging. In mice that had laser cuts for about a month, impacts on tissue architecture and cellular composition near the cuts were histologically examined. Lastly, the impact of cuts placed in the motor cortex on success in a skilled forelimb reaching/grasping task was examined. Mice were broken into three groups: sham surgery (*n* = 6) (just a craniotomy), regular ablation (*n* = 6) (laser cuts were placed in caudal forelimb motor cortex), and severing ablation (*n* = 7) (laser cuts in the same place, but with the addition of a full ablated layer at the bottom of the cylinder, thereby severing vertical cortical connections). In the task, mice learned to grasp a sugar pellet through a narrow opening, and changes in their success after treatment were assessed. Sham surgery mice repeated the experiment after being given a photothrombotic stroke of similar size to the laser ablated region, as a positive control. For the seizure propagation and grasping task experiments, group sizes were determined from power analysis based on results from pilot experiments (see Supporting Information).

## Conflict of Interest

The authors declare no conflict of interest.

## Author Contributions

S.L., T.H.S., and C.B.S. performed conceptualization. S.L., D.A.R., R.E.R., M.Z., N.N., O.B., T.H.S., and C.B.S. performed methodology. S.L., D.A.R., R.M., A.H., J.R., T.L.S., and O.B. performed the investigation. D.A.R. Software. S.L., D.A.R., and C.B.S. performed visualization. S.L., D.A.R., T.L.S., L.J., N.N., and C.B.S. performed formal analysis. M.Z., T.H.S., N.N., and C.B.S. performed funding acquisition. T.H.S. and C.B.S. performed project administration. S.L. and D.A.R. wrote–original draft. S.L., D.A.R., R.M., A.H., T.L.S., L.J., J.R., R.E.R., M.Z., N.N., O.B., T.H.S., C.B.S. wrote–reviewed and edited.

## Supporting information

Supporting Information

Supplemental Movie 1

Supplemental Movie 2

Supplemental Movie 3

Supplemental Movie 4

## Data Availability

The data that support the findings of this study are openly available in the Cornelll eCommons at https://doi.org/10.7298/dwp4-3p52.
